# Windbreak and airflow performance of different synthetic shrub designs based on wind tunnel experiments

**DOI:** 10.1371/journal.pone.0244213

**Published:** 2020-12-28

**Authors:** Xia Pan, Zhenyi Wang, Yong Gao, Zhengcai Zhang, Zhongjv Meng, Xiaohong Dang, Liqiang Lu, Jiahuan Chen

**Affiliations:** 1 College of Desert Control Science and Engineering, Inner Mongolia Agricultural University, Hohhot, Inner Mongolia Autonomous Region, China; 2 Wind Erosion Key Laboratory of Central and Government, Hohhot, Inner Mongolia Autonomous Region, China; 3 Key Laboratory of Desert and Desertification, Northwest Institute of Eco-Environment and Resources, Chinese Academy of Sciences, Lanzhou, China; 4 National Positioning Observation Research Station of Hangjin Desert Ecosystem, Ordos, Inner Mongolia Autonomous Region, China; Centro de Investigacion Cientifica y de Educacion Superior de Ensenada Division de Fisica Aplicada, MEXICO

## Abstract

Wind erosion has gained increasing attention as one of the most serious global ecological and environmental threats. Windbreaks are effective at decreasing wind erosion by reducing wind speed to protect crops, livestock, and farmsteads, while providing wildlife habitats. Synthetic shrubs can act as novel windbreaks; however, there is limited knowledge on how their design affects wind speed. This study determined the protective effects (airflow field and sheltering efficiency) based on the design of synthetic shrubs in a wind tunnel. Broom-shaped synthetic shrubs weakened the wind speeds mainly at the middle and upper parts of the shrubs (5–14 cm), while for hemisphere-shaped shrubs this effect was greatest near their bases (below 4 cm) and least in the middle and upper parts (7–14 cm). Spindle-shaped synthetic shrubs provided the best reduction effect in wind range and strength. Moreover, the wind speed reduction ratio decreased with improved wind speeds and ranged from 26.25 cm (between the second and third rows) to 52.5 cm (after the third row). These results provide strong evidence that synthetic shrubs should be considered to decrease wind speed and prevent wind erosion.

## Introduction

Wind erosion is an ecological and environmental issue of global concern, with many adverse effects such as damage to infrastructure, economic loss, increased regional poverty, and social instability [1,2]. Desertification affects 32% of the world’s population, 67% of countries, and 40% of the land area, making it a serious threat. Reducing the rate of land degradation was prioritized in 2007 through the Action Plan to Combat Desertification (APCD) [3]. Many countries have made progress in desertification control science through the United Nations Convention to Combat Desertification (UNCCD) [4,5]. China has initiated large, costly projects (accounting for 0.024% of China’s annual GDP) to combat desertification and sandstorms by increasing vegetation coverage [6]. China’s ecological protection projects are considered the most ambitious mega-afforestation engineering schemes in human history [7–10]. Since 1990, the area of human planting and aerial planting have been applied on 24.7 and 12.8 million hectares in China, respectively [11].

A windbreak is generally defined as a natural vegetative barrier against wind speed [12–14]. They are widely used in semiarid, arid, cold, and coastal environments to prevent wind erosion [15–17]. Since the 1940s, windbreaks have undergone systematic study to find the optimal windbreak—one that yields optimal protection at minimum cost [18]. Gandemer [19] and Perera [20] pointed out that the geometric shape of a single windbreak and the row spacing also affect the airflow distribution of windbreaks. However, their optimal design is still unclear [21–26]; they can comprise a single element or a system that, through their presence in the airflow, reduces the effects of the wind both in the immediate vicinity and within a given windward and leeward distance [27–29]. The efficiency of a windbreak is not only affected by geometric factors such as windbreak shape, height, width, and row spacing [27,30–32], but also by wind speed and surface roughness [33,34]. Clarifying the optimal characteristics would allow us to suggest an appropriate windbreak for any given application [27,35].

Studies in arid and semiarid areas have demonstrated that vegetation windbreaks are one of the most effective methods for reducing the hazards of wind erosion [36–38]. However, drought is a limiting factor for plant growth in deserts [39,40]. Degradation or loss of vegetation cover may further result in high environmental and economic costs by triggering sandstorms [35,41]. Drought stress can disturb plant-moisture relations and reduce moisture use efficiency in plants by decreasing stem elongation, root propagation, and leaf size. Most importantly, drought frequency and severity are predicted to worsen [42,43]. Extensive research has shown that wind speed characteristics and the protective effect of vegetation windbreaks depend on their geometric design, including height, length, width, porosity, opening size, distribution, and geometric shape [44]. Geometric design (also referred to here as “design” or “configuration”) is the main structural characteristic affecting the wind proofing efficiency of windbreaks [23,44]. Based on this, synthetic shrubs, which are three-dimensional, tangible, and flexible structures, are designed and used as alternatives to traditional (natural) windbreaks with short lifespans and high water consumption [27,45]. Synthetic shrubs are easy to install in extreme environmental conditions and are not affected by seasons. The specialized materials used in synthetic shrubs can be recycled, and they can have visual appeal.

In this study, the airflow fields and windbreak characteristics of synthetic shrubs consisting of artificial plants of different designs were studied in detail, based on a series of wind tunnel experiments with artificial plants that simulated the geometric shape of *Nitraria tangutorum*, a dominant shrub species in arid northern China.

## Methods

### Wind tunnel experiment setup

**Fig 1:**
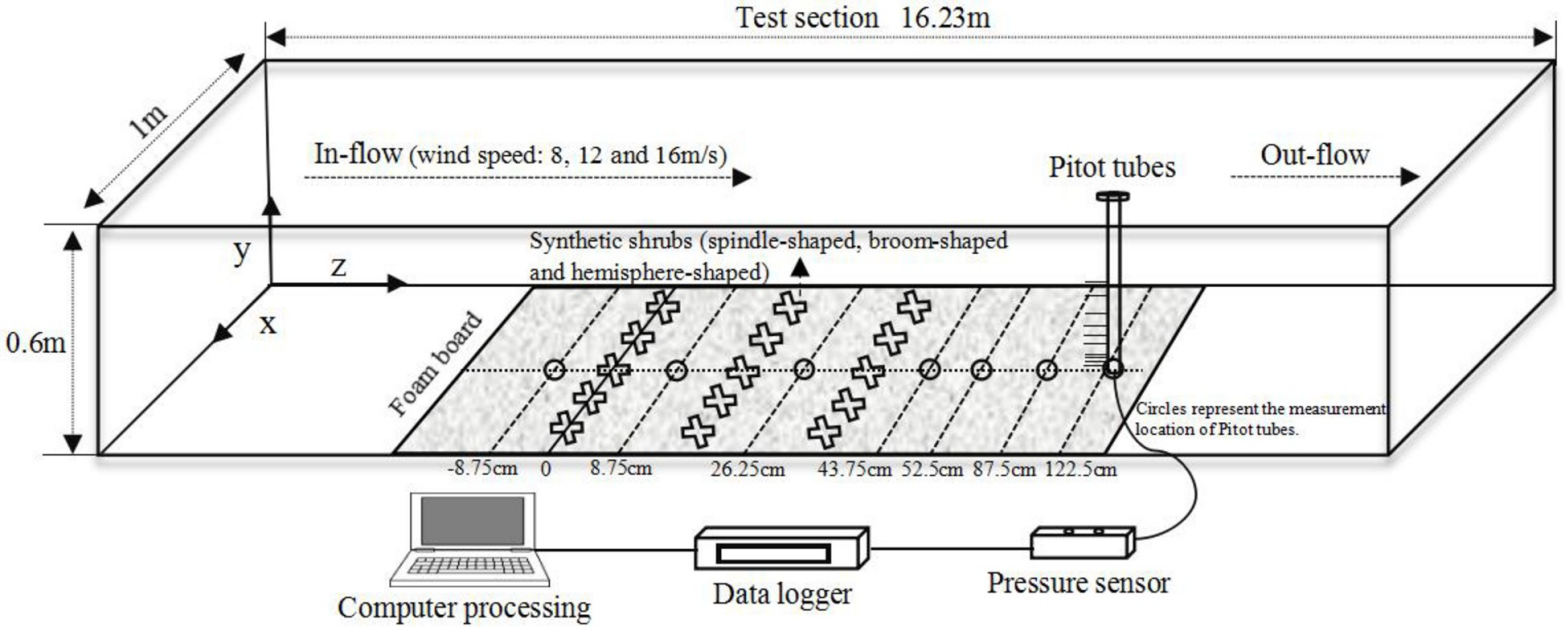
Test section of the wind tunnel experiments.

The wind tunnel experiments were conducted at the Key Laboratory of Desert and Desertification of the Chinese Academy of Sciences, Lanzhou, China [39]. The wind tunnel consisted of six sections: 1) air inflow, 2) flow contraction, 3) outflow diffusion section, 4) impeller, 5) test section, and 6) flow stabilization. The tunnel used was a non-circulating blow-type, with a speed range capability of 1–40 m/s (turbulence intensity < 0.4%). It was 37.8 m long with a test section of 16.2 m (length) × 0.6 m (height) × 1.0 m (width) (Fig 1). The thickness of the boundary layer in the test section was typically more than 120 mm, and the wind speeds were continuously adjustable.

### Structure and characteristics of synthetic shrubs

Before the wind tunnel experiments, we measured the geometric parameters of annual *Nitraria tangutorum*, including height, canopy dimension and porosity, and frontal area. The field measurements were performed in Dengkou County, Bayannur City, Inner Mongolia Autonomous Region, China (Table 1). Canopy dimensions comprised length diameter × width diameter of the leaf area of a single plant. The canopy porosity and frontal area were estimated by using an unsupervised classification analysis of canopy photographs using ERDAS IMAGINE 9.2 software (https://www.hexagongeospatial.com). Pixel numbers within a predetermined range of gray were counted, and the scaling relationship between pixel size and length was applied to calculate the frontal area. The synthetic shrubs used in this study were constructed at a reduced size but scaled in proportion (at 1:4) to the geometric morphology of field plants.

**Table 1:** Geometric parameters for field and synthetic shrubs of *Nitraria tangutorum*.

Shrubs		Height (cm)	Canopy dimension (cm)	Canopy porosity (%)	Frontal area (m^2^)
**Field shrubs**		89±11	70 (length) × 75 (width)	42–64	0.055–0.074
**Synthetic shrubs**	Hemisphere-shaped	22	15 (length) × 17.5 (width)	51	0.059
	Spindle-shaped	22	17.5 (length) × 15 (width)	47	0.052
	Broom-shaped	22	16 (length) × 16 (width)	55	0.062

**Fig 2:**
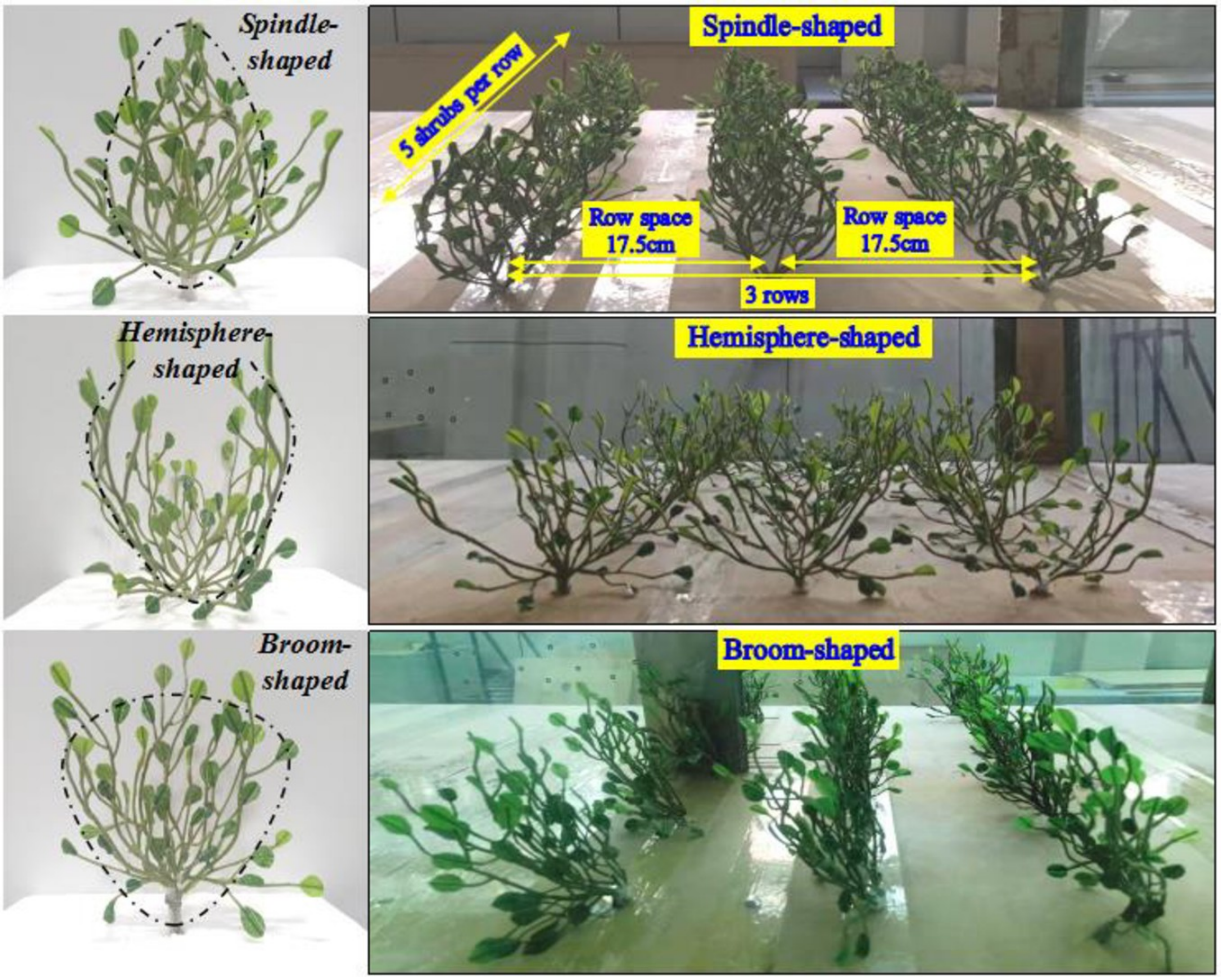
Configuration of synthetic shrubs (left) and arrangement in the test section of the wind tunnel (right).

Synthetic shrubs with leaves mimicking those of *Nitraria tangutorum* were constructed from a new type of polymerized wind- and ultraviolet-resistant material. Compared with the traditional materials of windbreaks with a lifespan of 3–5 years, the synthetic shrubs can last longer than 15 years. Besides the main trunk, the synthetic shrubs had 8–10 main branches, each containing 10–15 flat and obovate leaves. The latter were 3 cm long, 1.5 cm wide, and 0.1–0.2 mm thick. The shrubs were fixed upright by their main trunks in the test section of the wind tunnel before commencement of the experiments. The “roots” which held the rest of the synthetic plant secure were cemented below the wind tunnel. Only the branches and leaves were exposed in the test section, with plants being evenly dispersed as follows: the overall height of the shrubs was 22 cm, with 17.5 cm within the wind tunnel test section, and the remaining 4.5 cm below a foam board was connected to the roots. To ensure that the synthetic shrubs stood upright, the trunk and branches were constructed from steel wire wrapped in plastic. The shrubs had flexibility comparable to field plants. Based on the features of branches and leaves of *Nitraria tangutorum* and the characteristic forms of desert shrubs, three configurations of synthetic shrubs were constructed: spindle-shaped, broom-shaped, and hemisphere-shaped. The three configurations are shown in Figs 1 and 2.

Three rows of synthetic shrubs were fixed in place on a 1 m wide foam board in the test section of the wind tunnel (Figs 1 and 2). Each row included five shrubs evenly placed on the foam board. The distance between the rows and between the shrubs within each row was 17.5 cm, equal to the height of the shrubs in the test section. The wind tunnel experiment was conducted three times, once for the spindle-, broom-, and hemisphere-shaped shrub design, respectively.

### Measurements of wind speed

Wind speeds of 8, 12, and 16 m/s at different heights (0.2, 0.5, 1, 2, 4, 8, 12, 16, 20, and 24 cm) were measured using Pitot tubes along the central axis of the wind tunnel. The first row of synthetic shrubs was set as the origin (0 cm), and the distances from the origin to the seven measurement locations of Pitot tubes were -8.75 (the windbreak front), 8.75, 26.25 (within the windbreak), 43.75, 52.5, 87.5, and 122.5 cm (downwind of windbreak), respectively (Fig 1). Finally, wind speeds were measured continuously based on the wind pressure signals using a microdifferential-pressure sensor with FSKX-10. The wind speed at each measurement point was measured for 60 s. The resolution of the Pitot tubes was 1 s.

### Data analysis

Three wind speeds of 8, 12, and 16 m/s were generated at a height of 10 cm. Instantaneous wind speeds were calculated every 0.6 s at each measurement point, and the average values were recorded as U. Wind speeds were then measured again at the same heights without the synthetic shrubs in place, and the mean values were calculated as U_0_. Finally, the reduction ratio of the wind speed ((*U*_0_−*U*)/*U*)×100% was calculated to evaluate the windbreak efficiency. Contour figures were developed based on the Kriging interpolation using version 14.0 of the Surfer software (https://www.goldensoftware.com/). Other graphs were made in version 8.0 of the OriginPro software (https://www.originlab.com/).

## Results and analysis

### Distribution characteristics of the airflow field in synthetic shrubs

The airflow field represents the distribution characteristics of the wind speed in specific areas. The magnitude and degree of change of wind speed can be demonstrated based on the contour of wind speed. The darker the color, the smaller the wind speed; furthermore, the denser the contour line, the greater the change in airflow.

**Fig 3:**
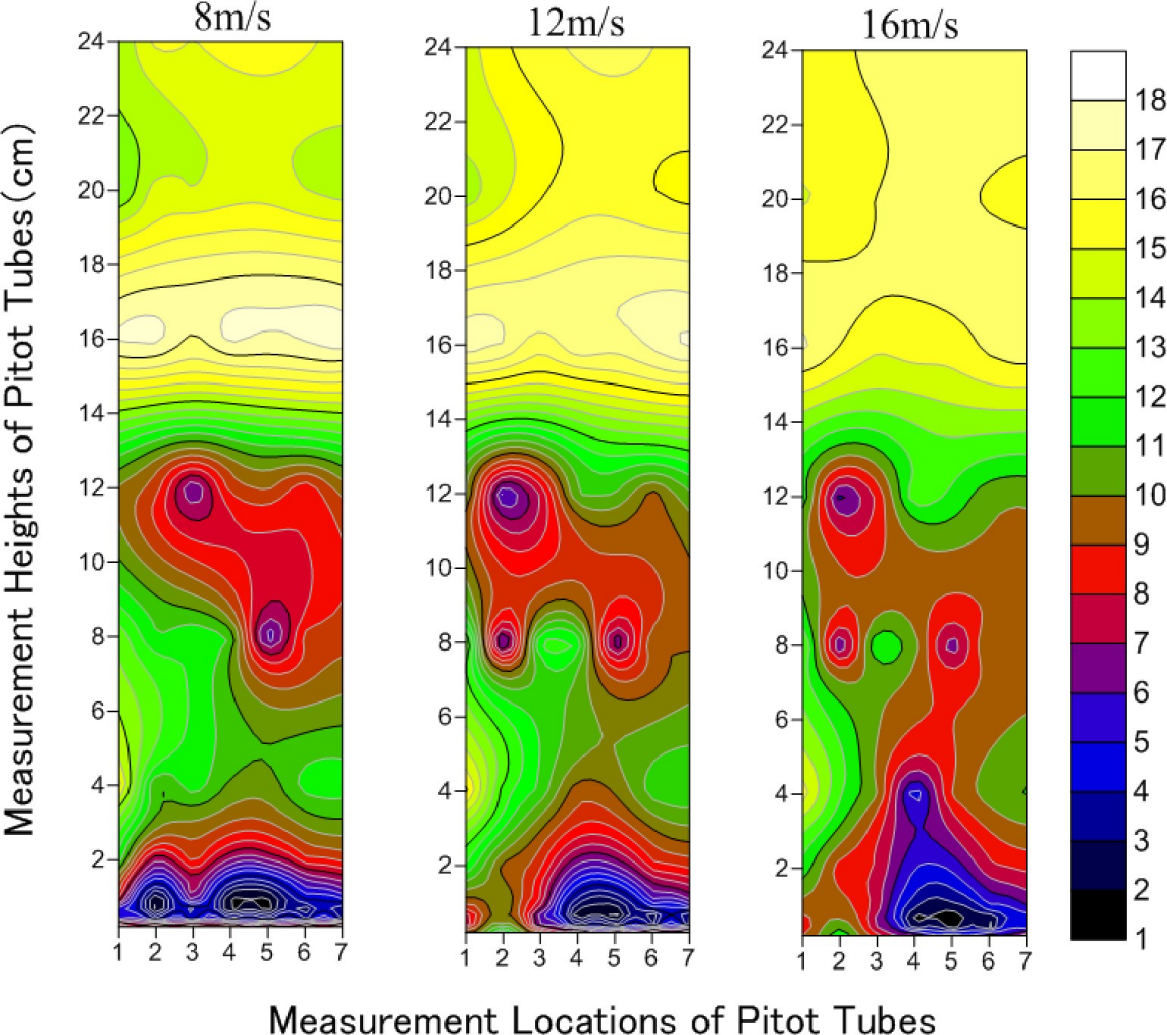
Distribution characteristics of airflow field in hemisphere-shaped synthetic shrubs under wind speeds of 8, 12 and 16 m/s. (Numbers 1–7 of horizontal axis correspond to -8.75 (the windbreak front), 8.75, 26.25 (within the windbreak), 43.75, 52.5, 87.5 to 122.5 cm (downwind of windbreak), respectively).

The airflow field distribution characteristics for hemisphere-shaped synthetic shrubs under wind speeds of 8, 12, and 16 m/s are presented in Fig 3. There was a distinctly lower wind speed (shown in blue and red) below 14 cm height. Measurements for the Pitot tubes were the same height as the synthetic shrubs. Since the total height of the synthetic shrubs in the test section was 17.5 cm, it could be deduced that the shrubs with this design had a significant effect on the reduction of wind speed. There were two obvious zones where the wind strength was weakened, namely below 4 cm (near the base) and at 7–14 cm (in the middle and upper parts of the synthetic shrubs). The weakening of wind strength was more pronounced near the base. However, the range of weakening of wind strength was lower than in the middle and upper parts. At 8 m/s, the weakened range of wind strength below 4 cm (near the base) was evenly distributed from -8.75 cm (the windbreak front) (1 horizontal axis) to 122.5 cm (downwind of windbreak) (7 horizontal axes). On the other hand, there was an obvious backward-shifting trend from 26.25 cm (within the windbreak) (3 horizontal axes) to 122.5 cm (downwind of windbreak) (7 horizontal axes) of the weakening area of wind strength below 4 cm (near the base) at 12 m/s and 16 m/s. Because synthetic and field shrubs have comparable bendability, they have similar capacities to buffer wind. Therefore, when the wind speed was 8 m/s, the airflow distribution near the base was more uniform. However, the shrubs did not effectively block the strong wind, at speed 16 m/s. Thus, the wind speed weakening zone retracted as the wind speed increased. When the wind speed was relatively high (16 m/s), the horizontal location of wind speed weakening was concentrated beyond 26.25 cm (within the windbreak) (3 horizontal axes).

**Fig 4:**
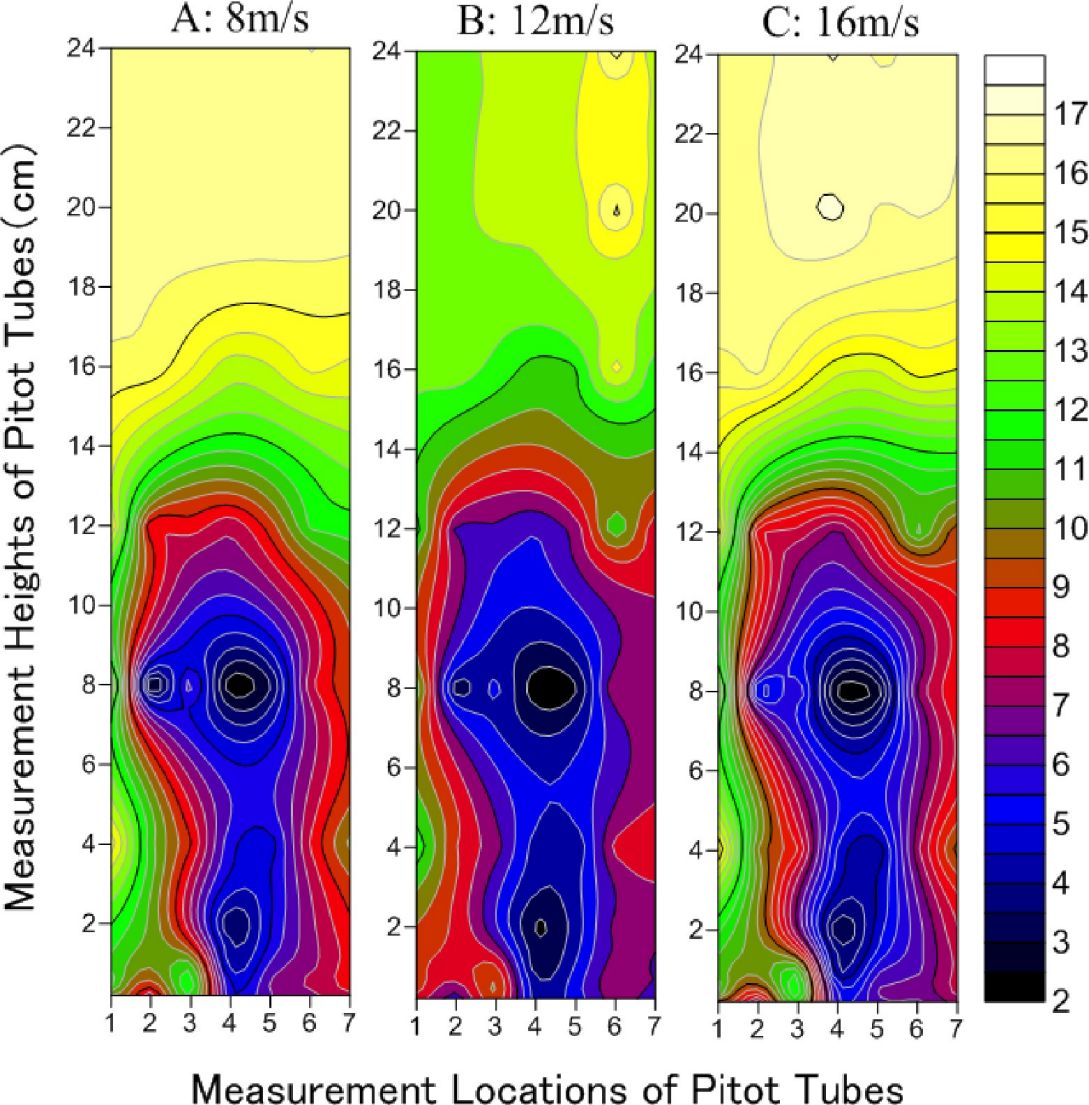
Distribution characteristics of airflow field in spindle-shaped synthetic shrubs under wind speeds of 8, 12 and 16 m/s. (Numbers 1–7 of horizontal axis correspond to -8.75 (the windbreak front), 8.75, 26.25 (within the windbreak), 43.75, 52.5, 87.5 to 122.5 cm (downwind of windbreak), respectively).

Distribution characteristics of the airflow field for the spindle-shaped synthetic shrubs under wind speeds of 8, 12, and 16 m/s differed markedly from those of the hemisphere-shaped shrubs (Fig 4). There were two continuous zones of weakening wind strength, specifically, at Pitot tube height below 5 cm, and between 5 and 14 cm. Two relatively steady airflow zones (shown in dark color) were formed inside the two continuous weakening zones of wind speed. The range and strength of reduced wind speed were more uniform for spindle-shaped than for hemisphere-shaped synthetic shrubs. Moreover, for the reduced wind speed, the horizontal measurement locations of the Pitot tubes ranged mainly from 43.75 cm to 122.5 cm (downwind of windbreak) (4–7 horizontal axes), behind the third row of shrubs, and below 5 cm. For heights of 5–14 cm, the range of reduced wind speed was wider than that below 5 cm. Both the range of wind speed reduction and change in airflow were greater at 16 m/s wind speed, as shown by the denser isolines. The isolines where the wind speed decreased to 12 m/s were the sparsest, but also the airflow was more stable at that point. The trend of wind speed change at 8 m/s was similar to that at 16 m/s, but the airflow was more stable, as shown by the relatively sparse isolines in comparison with those at 16 m/s. The effective height of wind speed weakening for the spindle-shaped synthetic shrubs was below 14 cm.

**Fig 5:**
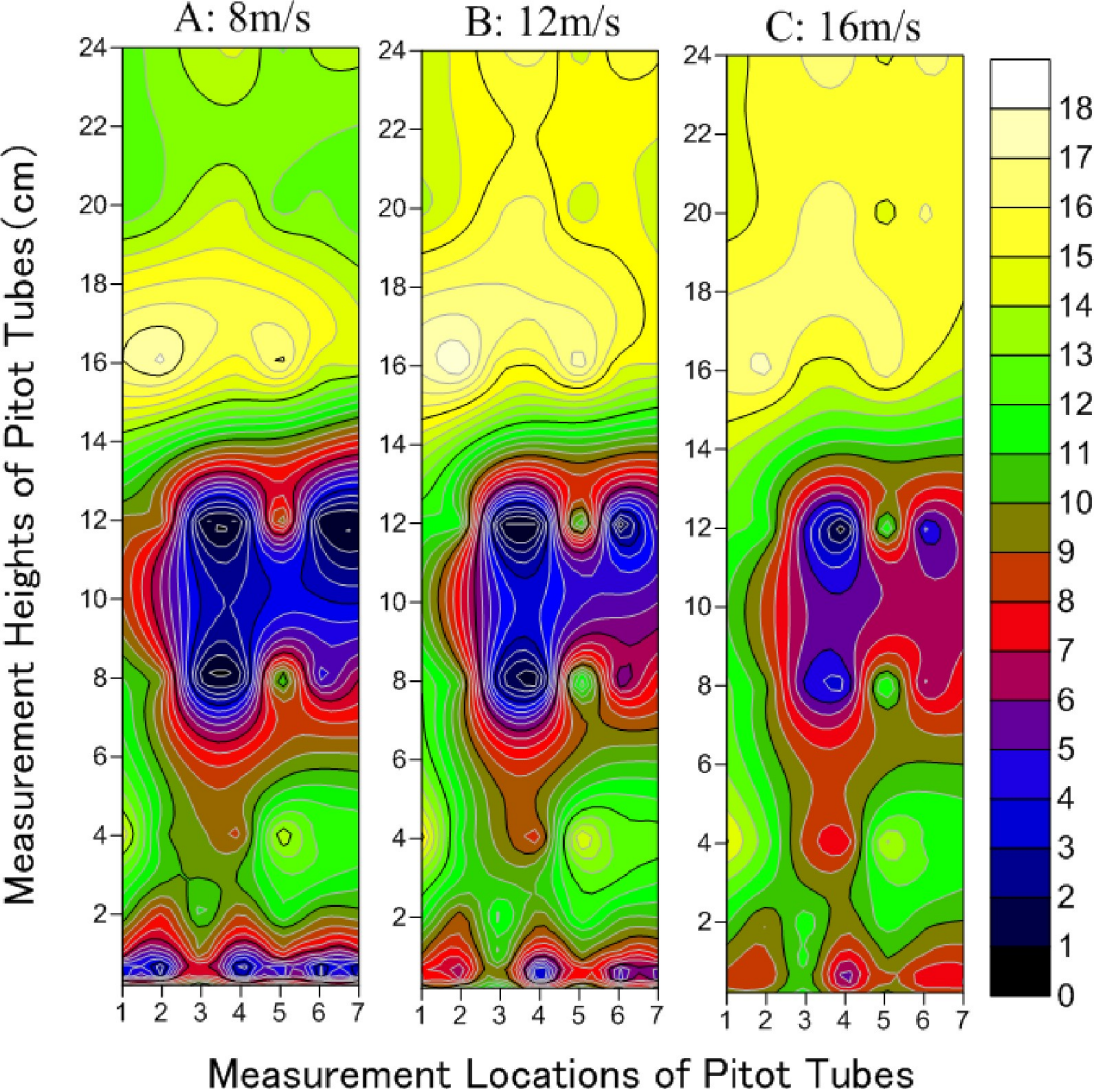
Distribution characteristics of airflow field in broom-shaped synthetic shrubs under wind speeds of 8, 12 and 16 m/s. (Numbers 1–7 of horizontal axis correspond to -8.75 (the windbreak front), 8.75, 26.25 (within the windbreak), 43.75, 52.5, 87.5 to 122.5 cm (downwind of windbreak), respectively).

Fig 5 shows that for the broom-shaped synthetic shrubs, at heights below 2 cm, the zone of reduced wind speed ranged from -8.75 cm to 122.5 cm (from the windbreak front to downwind of windbreak) (1–7 on horizontal axes). For 5–14 cm at the middle and upper parts of these shrubs, the zone of reduced wind speed ranged from 8.75 cm to 122.5 cm (from within the windbreak to downwind of windbreak) (2–7 on horizontal axes). Similar to the hemisphere-shaped synthetic shrubs, there were continuous zones of reduced wind speed, and the vertical measurement heights of Pitot tubes were below 2 cm near the base, and between 5 and 14 cm at the middle and upper parts of the broom-shaped synthetic shrubs. This shows that the weakening effect of the broom-shaped synthetic shrubs on wind strength was mainly in the middle and upper parts at 5–14 cm. For the broom-shaped synthetic shrubs, the wind speeds were high at 2–5 cm of the vertical measurement, which indicates that there was no obvious weakening effect on wind speed. Moreover, the weakening strength and range of the shrubs at a wind speed of 16 m/s were significantly less, indicating that the broom-shaped synthetic shrubs are not suitable for use in environments where the wind speed is greater than 16 m/s. The range of wind speed reduction for velocities of 8 m/s and 12 m/s were not significantly different, but the effect of wind speed reduction below 2 cm weakened.

### Effects of synthetic shrub design on wind speed

**Fig 6:**
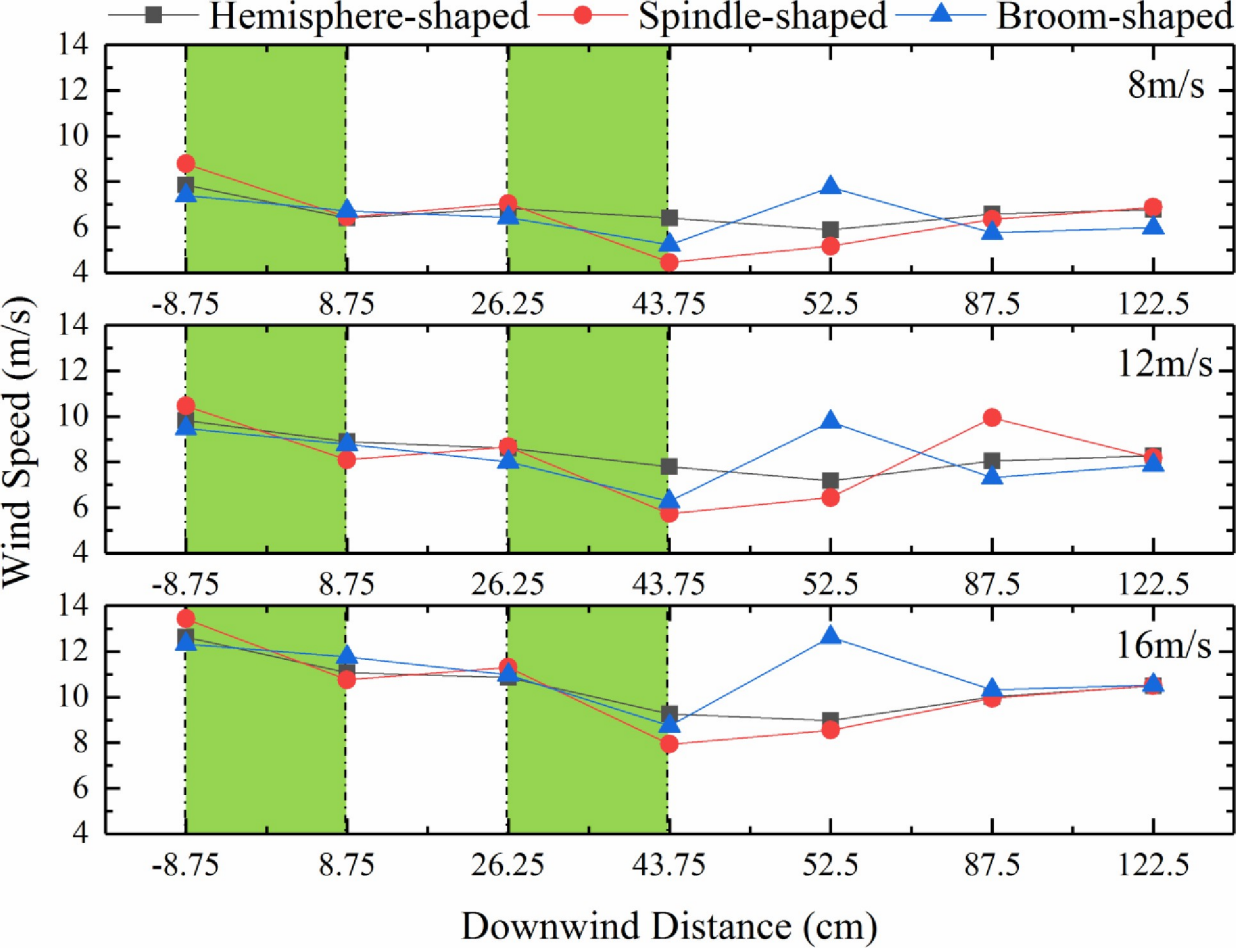
Effect of synthetic shrub design on wind speed. (The green shading represents an obvious downward trend in wind speed).

The wind speeds at the horizontal measurement locations of the synthetic shrubs from -8.75 cm to 8.75 cm (from the windbreak front to within the windbreak) (1–2 on horizontal axes) and from 26.25 cm to 43.75 cm (from within the windbreak to downwind of windbreak) (3–4 on horizontal axes) showed a downward trend (green shading in Fig 6). The vertical weakening height of the three forms of synthetic shrubs on the wind speed was within 4.5–13.5 m/s. The wind speeds were weakened at distances from 8.75 cm (within the windbreak) (2 horizontal axes) to 43.75 cm (downwind of windbreak) (4 horizontal axes) because the synthetic shrubs had a blocking effect (Fig 6). Downwind of the shrub, the wind speed increased (beyond 52.5 cm (downwind of windbreak) (5 horizontal axes)) and with increasing distance (87.5 cm (downwind of windbreak) (6 horizontal axes) and 122.5 cm (downwind of windbreak) (7 horizontal axes)) it decreased further (Fig 6). Therefore, the wind speed of the synthetic broom-shaped shrubs at 52.5 cm (downwind of windbreak) (5 horizontal axes) under 8 m/s, 12 m/s, and 16 m/s conditions first increased and then decreased. In addition, the wind speeds for spindle-shaped shrubs at 87.5 cm (downwind of windbreak) (6 horizontal axes) at 12 m/s also showed a sudden increase followed by a decrease. Moreover, the wind speeds at 8.75 cm (within the windbreak) (2 horizontal axes) and 26.25 cm (within the windbreak) (3 horizontal axes) decreased significantly compared to -8.75 cm (the windbreak front) (1 horizontal axis), indicating that the synthetic shrubs had a significant reduction effect on wind speed. The wind speed weakening was stronger between the second row at 26.25 cm (within the windbreak) (3 horizontal axes) and the rear row at 43.75 cm (downwind of windbreak) (4 horizontal axes) for both spindle-shaped and broom-shaped shrubs, while for hemisphere-shaped shrubs between 8.75 cm (within the windbreak) (2 horizontal axes) and 52.5 cm (downwind of windbreak) (5 horizontal axes). The wind speeds were lowest for the spindle-shaped and broom-shaped shrubs at 43.75 cm (downwind of windbreak) (4 horizontal axes), and for the hemisphere-shaped shrubs at 52.5 cm (downwind of windbreak) (5 horizontal axes), indicating that the wind speed weakening effect of the synthetic shrubs was the most obvious. The wind speed weakening effect for the hemisphere-shaped shrubs was significant, although not as strong as that for the spindle-shaped shrubs, which was the best of the three designs.

### Shelter efficiency based on synthetic shrub design

**Fig 7:**
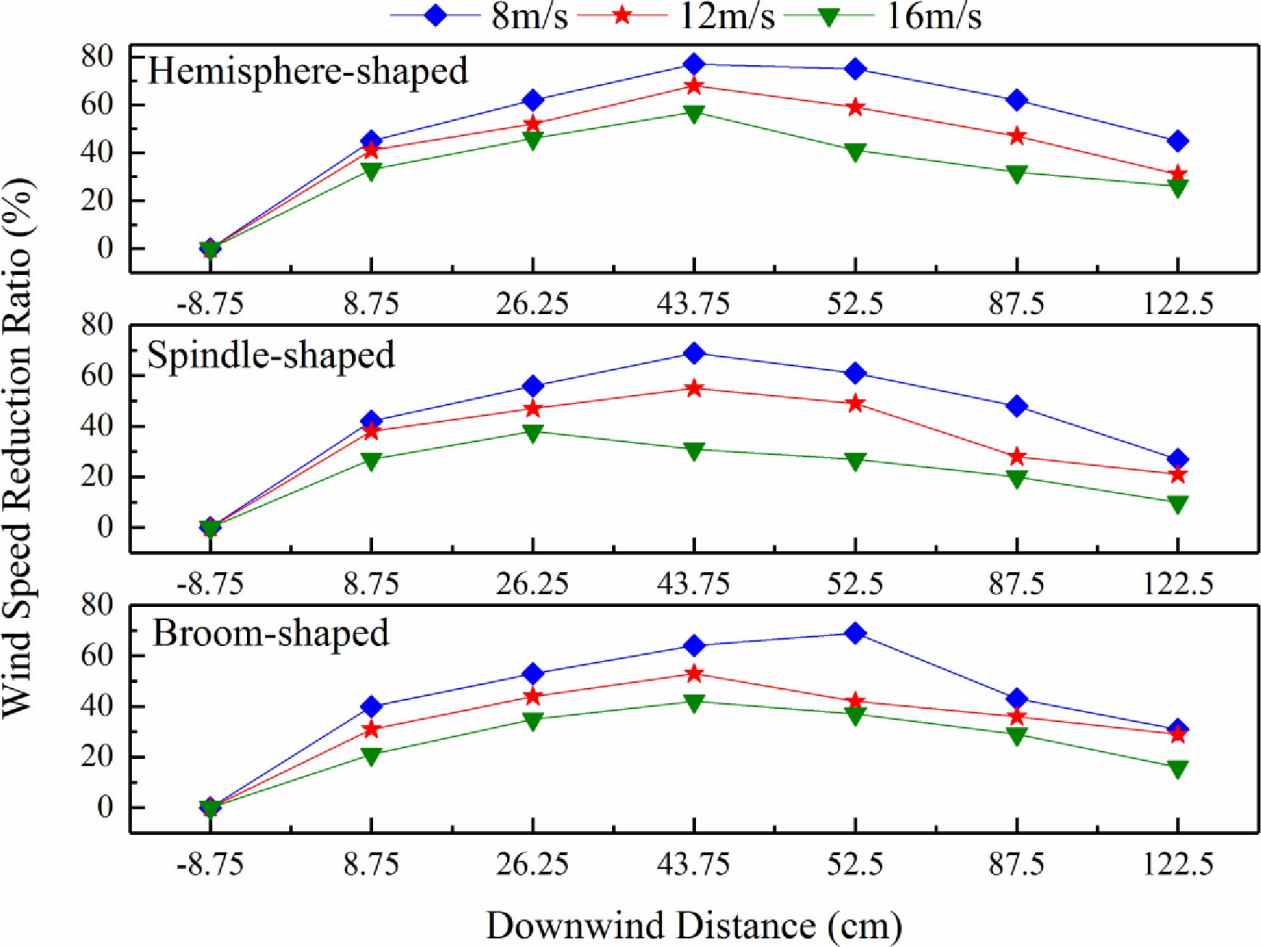
Effects of synthetic shrub design on wind speed reduction ratio.

The effects of the design of synthetic shrubs on the wind speed reduction ratio were analyzed (Fig 7). The reduction ratio for synthetic shrubs with different designs at wind speeds of 8 m/s, 12 m/s, and 16 m/s was greatest at 8 m/s, indicating that it decreased as the speed increased. Moreover, it showed a trend of first increasing and then decreasing. For hemisphere-shaped synthetic shrubs, this ratio increased when the horizontal measurement locations of the Pitot tubes were from -8.75 cm (the windbreak front) (1 horizontal axes) to 43.75 cm (downwind of windbreak) (4 horizontal axes) due to the blocking effect of the shrubs. The wind speed reduction ratio of hemisphere-shaped synthetic shrubs reached a maximum when the horizontal measurement locations of the Pitot tubes were 43.75 cm (downwind of windbreak) (4 horizontal axes), beyond which the ratio showed a downward trend. For the spindle-shaped synthetic shrubs under a wind speed of 16 m/s, the reduction ratio reached a maximum at 26.25 cm (within the windbreak) (3 horizontal axes). There was an upward trend from -8.75 cm (the windbreak front) (1 horizontal axes) to 8.75 cm (within the windbreak) (2 horizontal axes) for the broom-shaped shrubs at wind speeds of 12 m/s and 16 m/s. The reduction ratio increased at distances from -8.75 cm (windbreak front) (1 horizontal axis) to 52.5 cm (downwind of windbreak) (5 horizontal axes) under a wind speed of 8 m/s and reached a maximum at 52.5 cm (downwind of windbreak) (5 horizontal axes). The highest values of wind speed reduction ratio for the three synthetic shrub designs were reached at distances ranging from 26.25 cm (within the windbreak) (3 horizontal axes) to 52.5 cm (downwind of windbreak) (5 horizontal axes).

## Discussion

The airflow fields around different designs of synthetic shrubs are important in determining the windbreak efficiency. The effects of windbreaks on wind speed attenuation depend on both the internal (e.g., density, shape, and number of branches) and external (e.g., length, width of windbreaks, and number of rows) structures [13]. Based on the wind tunnel experiments conducted in this study, we found that the zone of weakened wind strengths resulting from the spindle-shaped synthetic shrubs was both wider and more uniform than those of the other designs. There was a greater reduction in wind speeds near the base of hemisphere-shaped shrubs than for the other designs, but the weakening zone was smaller than in the middle and upper parts. However, for the broom-shaped shrubs, significant wind strength weakening occurred in the middle and upper parts (at 8–13 cm). Liu et al. [46] demonstrated that the variation in the wind acceleration zone was affected by the vegetation shape. Cornelis et al. [47] tested the effects of one-, two-, and three-row windbreaks on wind speed reduction based on a wind tunnel experiment. They found that the two- and three-row windbreaks were more efficient. Although our study highlighted the effects of different synthetic shrub designs on the airflow field and windbreak efficiency using three shrub rows, the wind speeds at distances beyond the middle of the second and third rows (from 8.75 cm to 122.5 cm) showed a downward trend compared to the front of the first row (-8.75 cm) (Fig 6). Furthermore, the wind speed reached its lowest level at 43.75 cm (the first measuring point behind the third row) for wind speeds of 8, 12, and 16 m/s (Fig 6).

The wind profiles demonstrated that the wind attenuation effect of the shrubs is closely related to the height of the plant’s foliage, where the wind-reducing influence of the plant frontal area is improved, and speed reduction is increased [48]. The wind speed increases above the vegetated layer because of the regional reduction in turbulence and shear stress [49,50]. These findings align with our results, in that the wind speed above the synthetic shrubs (> 14 cm) was higher (Figs 3–5). Zhang et al. [51] showed that the daily average windward wind speed at 2 m was reduced by 18%, 31%, and 66% in the shelterbelt center, 66% behind the shelterbelt, and 62% and 45% from the forest edge on the leeward side. The location of the greatest reduction ratio behind windbreaks marks the wind speed recovery zone, where the wind speed is the lowest [52]. However, Seguin [53] showed that at the local scale, the reduction was less effective for the second (and successive) downwind windbreaks than for the first windbreak. A windbreak slows down the average wind speed on a regional scale because it increases the terrain roughness. In this study, the wind speed weakening by synthetic shrubs of various designs was stronger in the distance range from 8.75 cm (the middle of the first and second rows) to 87.5 cm (the third measuring point behind the third row). A larger void (higher under-crown height) allows a higher ratio of wind penetration through windbreaks, leading to lower reduction efficiency above the height of 12 cm [54]. Fu et al. [55] showed that the wind speed could be significantly affected by vegetation morphology. Okin [56] and Leenders et al. [57] proposed triangular, rectangular, and semi-elliptical shapes for a “protective region”, where wind erosion is reduced or eliminated [55].

Field and theoretical studies, as well as numerical simulations, indicate that the airflow around plants can be affected by many factors such as plant density and distribution (e.g., number of leaves and/or stems), porosity and height-to-width ratios [58,59], leaf morphology, uniform or non-uniform distribution of leaves above the bed, plant rigidity, degree of prostrate versus erect form, the distribution of leaves on the stem or trunk, and the height of the trunk or stem above the surface [60–62]. Further research is required to examine the effects of other impact factors of synthetic shrubs on wind speed to improve their protective effects. These findings are useful for the design and engineering of ecological protection.

## Conclusions

The zone of reduced wind speed from spindle-shaped shrubs was wider and more uniform than for the other two synthetic shrub shapes; furthermore, the reduction in wind strength was also greater. The effect of the broom-shaped synthetic shrubs was to weaken the wind strength mainly in the middle and upper parts of the synthetic shrubs (5–14 cm). For the hemisphere-shaped shrubs, the weakening of wind strength was more effective near the base (below 4 cm), and less so in the middle and upper parts of the shrub (7–14 cm). Therefore, spindle-shaped synthetic shrubs provided the most effective weakening of the range and strength of the wind. The vertical weakening of wind speeds by the three synthetic shrub configurations ranged from 4.5 to 13.5 m/s. However, the wind speed reduction ratios for the three different synthetic shrub designs indicated that the ratio would decrease with increasing wind speed. The wind speed reduction ratio under wind speeds of 8, 12, and 16 m/s was greatest at distances ranging from 26.25 cm (between the second and third rows) to 52.5 cm (after the third row). These conclusions provide strong evidence that synthetic shrubs should be promoted for use to reduce wind speed and prevent wind erosion.

## Supporting information

S1 Data All data are fully available without restriction. All relevant data are within the manuscript and its supporting information files.
(XLSX)

## References

[1] Shen W, Li H, Sun M, Jiang J. Dynamics of aeolian sandy land in the Yarlung Zangbo River basin of Tibet, China from 1975 to 2008. Global and Planetary Change. 2012; 86: 37–44.

[2] Chen X, Duan Z, Tan M. Restoration Affect Soil Organic Carbon and Nutrients in Different Particle-size Fractions. Land Degradation & Development. 2016; 27: 561–572.

[3] Reynolds JF, Smith DMS, Lambin EF, TurnerII BL, Mortimore M, Batterbury SPJ, et al Global desertification: building a science for dryland development. Science. 2007; 316: 847–851. 10.1126/science.1131634 17495163

[4] Zang Y, Gong W, Xie H, Liu B, Chen H. Chemical sand stabilization: A review of material, mechanism, and problems. Environmental Technology Reviews. 2015; 4: 119–132.

[5] Safriel U. Land Degradation Neutrality (LDN) in drylands and beyond-where has it come from and where does it go. Silva Fennica. 2017; 51: 1–19.

[6] Wang G, Wang X, Wu B, Lu Q. Desertification and its mitigation strategy in China. Journal of Resources and Ecology. 2012: 3: 97–104.

[7] Ouyang ZY, Zheng H, Xiao Y, Polasky S, Liu JG, Xu WH, et al Improvements in ecosystem services from investments in natural capital. Science. 2016; 352: 1455–1459. 10.1126/science.aaf2295 27313045

[8] Hua FY, Wang XY, Zheng XL, Brendan F, Wang L, Zhu JG, et al Opportunities for biodiversity gains under the world’s largest reforestation program. Nat Commun. 2016; 7: 12717 10.1038/ncomms12717 27598524 PMC5025860

[9] Moore JC, Chen Y, Cui WY, Yuan WP, Dong WJ, Gao Y, et al Will China be the first to initiate climate engineering. Earth’s Future. 2016; 4: 588–595.

[10] Xu WH, Xiao Y, Zhang JJ, Yang W, Zhang L, Hull V, et al Strengthening protected areas for biodiversity and ecosystem services in China. Proc. Natl Acad. Sci. USA. 2017; 114: 1601–1606. 10.1073/pnas.1620503114 28137858 PMC5321011

[11] Li WH. Degradation and restoration of forest ecosystems in China. For. Ecol. Manage. 2004; 201: 33–41.

[12] Rosenberg NJ. Microclimate: The Biological Environment. Wiley, New York 1974.

[13] Torita H, Satou H. Relationship between shelterbelt structure and mean wind reduction. Agric. For. Meteorol. 2007; 145(3–4): 186–194.

[14] Cheng H, He W, Liu C, Zou X, Kang L, Chen T, et al Transition model for airflow fields from single plants to multiple plants. Agric. For. Meteorol. 2019; 266: 29–42.

[15] Skidmore EL. Wind erosion. Soil Erosion Research Methods. 1988.

[16] Munson SM, Belnap J, Okin GS. Responses of wind erosion to climate-induced vegetation changes on the Colorado Plateau. Proc. Natl. Acad. Sci. 2011; 108: 3854–3859. 10.1073/pnas.1014947108 21368143 PMC3053991

[17] Li GS, Qu JJ, Han QJ, Fang HY, Wang WF. Responses of three typical plants to wind erosion in the shrub belts atop Mogao Grottoes, China. Ecol. Eng. 2013; 57: 293–296.

[18] Dong ZB, Luo WY, Qian GQ, Wang HT. A wind tunnel simulation of the mean velocity fields behind upright porous fences. Agricultural and Forest Meteorology. 2007; 146(1–2): 82–93.

[19] Gandemer J. Wind shelters. J. Ind. Aerodyn. 1979; 4: 371–389.

[20] Perera MDAES. Shelter behind two-dimensional solid and porous fences. J. Wind Eng. Ind. Aerodyn. 1981; 8: 93–104.

[21] Raupach MR. Drag and drag partition on rough surfaces. Bound.-Layer Meteorol. 1992; 60: 375–395.

[22] Finnigan JJ. Turbulence in plant canopies. Annu. Rev. Fluid Mech. 2000; 32: 519–571.

[23] Cornelis WM, Gabriels D. Optimal windbreak design for wind-erosion control. J. Arid Environ. 2005; 61(2): 315–332.

[24] Neumeier U. Quantification of vertical density variations of salt-marsh vegetation. Estuar. Coast. Shelf Sci. 2005; 63(4): 489–496.

[25] Cao J, Tamura Y, Yoshida A. Wind tunnel study on aerodynamic characteristics of shrubby specimens of three tree species. Urban For. Urban Green. 2012; 11(4): 465–476.

[26] Hong Y, Kim D, Im S. Assessing the vegetation canopy influences on wind flow using wind tunnel experiments with artificial plants. J. Earth Syst. Sci. 2016; 125(3): 499–506.

[27] Wu X, Zou X, Zhou N, Zhang C, Shi S. Deceleration efficiencies of shrub windbreaks in a wind tunnel. Aeolian Res. 2015; 16: 11–23.

[28] Zhang Z, Dong Z, Li C. Wind regime and sand transport in China’s Badain Jaran Desert. Aeolian Research. 2015; 17: 1–13.

[29] Yang YY, Liu LY, Shi PJ, Zhao MD, Dai JD, Lyu YL, et al Converging effects of shrubs on shadow dune formation and sand trapping. Journal of Geophysical Research: Earth Surface, 2019; 124(7): 1835–1853.

[30] Zhou XH, Brandle JR, Takle ES, Mize CW. Estimation of the three-dimensional aerodynamic structure of a green ash shelterbelt. Agric. For. Meteorol. 2002; 111(2): 93–108.

[31] Brandle JR, Hodges L, Zhou XH. Windbreaks in the North American agricultural systems. Agrofor. Syst. 2004; 61: 65–78.

[32] Ma R, Wang J, Qu J, Liu H. Effectiveness of shelterbelt with a non-uniform density distribution. J. Wind Eng. Ind. Aerodyn. 2010; 98(12): 767–771.

[33] Banzhaf J, Leihner DE, Buerkert A, Serafini PG. Soil tillage and wind break effects on millet and cowpea: I. Wind speed, evaporation, and wind erosion. Agron. J. 1992; 84: 1056–1060.

[34] Zhang Z, Dong Z, Qian G, Wu G, Cui X. An Investigation into the Processes and Quantity of Dust Emissions over Gravel and Sand Deserts in North-Western China. Boundary-Layer Meteorol. 2017; 163(3): 523–535.

[35] Hesp PA, Dong Y, Cheng H, Booth JL. Wind flow and sedimentation in artificial vegetation: field and wind tunnel experiments. Geomorphology. 2019; 337: 165–182.

[36] William HS, Jane AR, Anne EH, Anne FC. On the Spatial Pattern of Soil Nutrients in Desert Ecosystems. Ecology. 1996; 77(2): 364±74.

[37] Xin RL, Feng YM, Hong LX, Xin PW, Ke CK. Long-term effects of revegetation on soil water content of sand dunes in arid region of Northern China. Journal of Arid Environments. 2004; 57(1): 1±16.

[38] Torres L, Abraham EM, Rubio C, Barbero-Sierra C, Ruiz-PeÂrez M. Desertification Research in Argentina. Land Degradation & Development. 2015; 26(5): 433±40.

[39] Liu Y, Liu Y. Converging effects of shrubs on shadow dune formation and sand trapping. Journal of Geophysical Research: Earth Surface. 2019; 124(7): 1835–1853.

[40] Liu BL, Qu JJ, Ning DH, Han QJ, Yin DY, Du PF. WECON: A model to estimate wind erosion from disturbed surfaces. CATENA. 2019; 172: 266–273.

[41] Chen YF, Cai QG, Tang HP. Dust storm as an environmental problem in north China. Environ. Manage. 2003; 32: 413–417. 10.1007/s00267-003-0042-1 14986891

[42] Boutraa T, Akhkha A, Al-Shoaibi AA, Alhejeli AM. Effect of water stress on growth and water use efficiency (WUE) of some wheat cultivars (Triticum durum) grown in Saudi Arabia. J. Taibah Univ. Sci. 2010; 3: 39–48.

[43] Yongrsquo L, Quanwen D, Zhiguo C, Deyong Z. Effect of drought on water use efficiency, agronomic traits and yield of spring wheat landraces and modern varieties in Northwest China. Afr. J. Agri. Res. 2010; 5: 1598–1608.

[44] Chen BY, Cheng JJ, Xin LJ, Wang R. Effectiveness of hole plate-type sand barriers in reducing aeolian sediment flux: Evaluation of effect of hole size. Aeolian Research. 2019; 38: 1–12.

[45] Zhang YH, Kang CZ, Liu SZ, Tang JN, Wei LY, Li JH. Windbreak effect of *Picea mongolica* farmland shelterbelt with different configuration. J. Desert Res. 2017; 37(5): 859–866.

[46] Liu CC, Liu YG, Guo K, Li GQ, Zheng YR, Yu LF, et al Comparative ecophysiological responses to drought of two shrub and four tree species from karst habitats of southwestern China. Trees, 2011; 25: 537–549.

[47] Cornelis WM, Gabriels D, Gryse SD, Hartmann R. The efficiency of vegetative windbreaks in combating wind erosion: simulation and scaling. Sécheresse. 2000; 11(1): 52–7.

[48] Miri A, Dragovich D, Dong ZB. Wind-borne sand mass flux in vegetated surfaces-Wind tunnel experiments with live plants. CATENA. 2019; 172: 421–434.

[49] Dupont S, Brunet Y. Coherent structures in canopy edge flow: a large-eddy simulation study. J. Fluid Mech. 2009; 630: 93–128.

[50] Dupont S, Bonnefond JM, Irvine MR, Lamaud E, Brunet Y. Long-distance edge effects in a pine forest with a deep and sparse trunk space: in situ and numerical experiments. Agric. For. Meteorol. 2011; 151: 328–344.

[51] Zhang H, Li F, Zhang T, Zhao L, Yasuhito S. Evaluation of ecological services of Populus simonii forest on Heerqin sandy land. Yingyong Shengtai Xuebao. 2003; 14(10): 1591–1596. 14986346

[52] Li X, Ma Y, Ma R, Zhang Y, Tang W, Yang J. Wind flow field and windproof efficiency of shelterbelt in different width. J. Desert Res. 2018; 38(5): 936–944.

[53] Seguin B. Rugosite du paysage et evapotranspiration potentielle a l’echelle regionale. Agricultural Meteorology. 1973; 11: 79–98.

[54] Van-Thuyet D, Van-Do T, Sato T, Hung TT. Effects of species and shelterbelt structure on wind speed reduction in shelter. Agroforest Syst. 2014; 88: 237–244.

[55] Fu LT, Fan Q, Huang ZL. Wind speed acceleration around a single low solid roughness in atmospheric boundary layer. Scientific Reports. 2019; 9: 12002 10.1038/s41598-019-48574-7 31427684 PMC6700104

[56] Okin GS. A new model of wind erosion in the presence of vegetation. J. Geophys. Res. 2008; 113: F02S10.

[57] Leenders JK, Sterk G, Van-Boxel JH. Modelling wind-blown sediment transport around single vegetation elements. Earth Surf. Proc. Land. 2011; 36(9): 1218–1229.

[58] Gillies JA, Nield JM, Nickling WG. Wind speed and sediment transport recovery in the lee of a vegetated and denuded nebkha within a nebkha dune field. Aeolian Res. 2014; 12: 135–141.

[59] Cheng H, Zhang K, Liu C, Zou X, Kang L, Chen T, Fang Y. Wind tunnel study of airflow recovery on the lee side of single plants. Agric. For. Meteorol. 2018; 263: 362–372.

[60] Gillies JA, Nickling WG, King J. Drag coefficient and plant form response to wind speed in three plant species: Burning Bush (Euonymus alatus), Colorado Blue Spruce (Picea pungens glauca.), and Fountain Grass (Pennisetum setaceum). J. Geophys. Res. 2002; 107(D24): 4760.

[61] Leenders JK, Van-Boxel JH, Sterk G. The effect of single vegetation elements on wind speed and sediment transport in the Sahelian zone of Burkina Faso. Earth Surf. Process. Landf. 2007; 32(10): 1454–1474.

[62] Dupont S, Bergametti G, Simoëns S. Modeling aeolian erosion in presence of vegetation. Geophys Res. Earth Surf. 2014; 119(2): 168–187.

